# ASD Symptom Severity in Adolescence of Individuals Diagnosed with PDD-NOS in Childhood: Stability and the Relation with Psychiatric Comorbidity and Societal Participation

**DOI:** 10.1007/s10803-015-2595-2

**Published:** 2015-09-22

**Authors:** A. Louwerse, M. L. J. M. Eussen, J. Van der Ende, P. F. A. de Nijs, A. R. Van Gool, L. P. Dekker, C. Verheij, F. Verheij, F. C. Verhulst, K. Greaves-Lord

**Affiliations:** Department of Child and Adolescent Psychiatry/Psychology, Erasmus MC - Sophia Children’s Hospital, Wytemaweg 8, 3015 CN Rotterdam, The Netherlands; Yulius, Organisation for Mental Health, Yulius Academy, Hellingen 21, 3311 GZ Dordrecht, The Netherlands

**Keywords:** Autism spectrum disorders (ASD), Pervasive developmental disorder-not otherwise specified (PDD-NOS), Comorbid psychiatric disorders, Follow-up, Autism Diagnostic Observation Schedule (ADOS-2), Symptom severity, Societal functioning

## Abstract

The current 7-year follow-up study investigated: (1) the stability of ASD severity, and (2) associations of ASD severity in adolescence with (a) childhood and concurrent psychiatric comorbidity, and (b) concurrent societal functioning. The Autism Diagnostic Observation Schedule (ADOS) and the Diagnostic Interview Schedule for Children were administered in childhood (ages 6–12) and in adolescence (ages 12–20) to 72 individuals with a pervasive developmental disorder-not otherwise specified (PDD-NOS). ADOS calibrated severity scores showed a large stability (*r* = .51). Psychiatric comorbidity in childhood and adolescence were not associated with ASD severity in adolescence. Mental health care use (87 %) and special education needs were high (71 %). Reevaluation of ASD severity and psychiatric comorbidity later in life seem useful when PDD-NOS is diagnosed in childhood.

## Introduction

In the past decades, the number of children diagnosed with autism spectrum disorder (ASD) increased fourfold (Duchan and Patel 2012). This increase in childhood diagnoses of ASD implies a growing public health issue, since individuals with ASD often use mental health care and special education, probably not only in childhood, but also later in life. Since children currently diagnosed with ASD will be transitioning to adolescence in the next decade, clarification of their future needs and possibilities is warranted for policy makers and all those involved in the care and education of children with ASD (Hus and Lord 2014; Orsmond et al. 2013).

ASD is generally considered a life-long disorder (APA 2000). However, studies that investigated the stability of ASD vary widely in the reported stability rates (Billstedt et al. 2005; Cederlund et al. 2008; Charman et al. 2005; Chawarska et al. 2007; Guthrie et al. 2012; Lord et al. 2006; Malhi and Singhi 2011; McGovern and Sigman 2005; Moore and Goodson 2003; Moss et al. 2008; Rondeau et al. 2010; Soke et al. 2011; Starr et al. 2003; Turner and Stone 2007; Woolfenden et al. 2012). This variation in results can be attributed to differences in sample characteristics, e.g. type of ASD classification and intelligence level. Firstly, regarding type of ASD diagnoses, some studies mainly included individuals with autistic disorder (AD, e.g. Charman et al. 2005; McGovern and Sigman 2005), while others included individuals with more broadly defined ASD, including Asperger’s syndrome (AS) and pervasive developmental disorder-not otherwise specified (PDD-NOS; e.g. Cederlund et al. 2008; Lord et al. 2006). From these studies it seems that AD is more stable across time (70–98 %; e.g. Kleinman et al. 2008; McGovern and Sigman 2005) than AS or PDD-NOS (17–100 %; e.g. Kleinman et al. 2008; Malhi and Singhi 2011; Rondeau et al. 2010). However, longitudinal studies up to adolescence or adulthood including individuals with more broadly defined ASD (i.e. AS or PDD-NOS) are sparse. Secondly, as for intelligence level, studies including individuals with ASD and a Full Scale Intelligence Quotient (FSIQ) below 70 (e.g. Kleinman et al. 2008; Malhi and Singhi 2011; McGovern and Sigman 2005) reported greater stability than studies including individuals with an FSIQ above 70 (Billstedt et al. 2005; Cederlund et al. 2008). Most previous longitudinal studies included individuals diagnosed with autism according to the DSM-III (i.e. infantile autism) and, in most studies, the participants also had a cognitive impairment. These individuals might not represent the individuals diagnosed with ASD in the last decade. Further research particularly including individuals with PDD-NOS diagnosed in the 21th century without a cognitive impairment is important, since these individuals might better represent the individuals currently diagnosed with ASD (CDC 2012; Fombonne 2009).

Previous research concerning the stability of ASD has been complicated by the ongoing debate on the conceptualisation of ASD. Over the past decades, diagnostic criteria changed and diagnostic methodologies altered, impacting findings on diagnostic stability. Changing the boundaries of a diagnostic category directly affects the number of individuals who remain to be part of this category. Therefore, a dimensional approach—examining autistic traits on a continuum—can be a valuable addition to studies on stability, since results are not affected by the boundaries set for a particular category that might be changed with new insights. Previous studies concerning ASD stability mostly used a categorical diagnostic approach. Thus, these studies could not benefit from standardized diagnostic tools that generate continuous scores and which can be used with individuals of all ages (Levy and Perry 2011; Volkmar and Reichow 2013). The recent development of the Autism Diagnostic Observation Schedule (ADOS-2) calibrated severity scores (CSS) now creates the possibility to examine stability of clinically observed ASD severity across development, since this continuous measure can be used to quantitatively compare the level of ASD symptom severity over time, thus investigating stability using a dimensional approach (Gotham et al. 2009, Hus and Lord 2014). These CSS provide the opportunity to compare ADOS scores longitudinally using different, age- and expressive language level appropriate modules. CSS are less influenced by age and cognitive ability compared to raw totals. Also, CSS are relatively independent of non-ASD-specific behavior problems (Gotham et al. 2009, Hus and Lord 2014). The developers of the ADOS and the subsequent CSS have replicated their findings in an independent sample, including modules one to three (Bal and Lord 2015). However, the external validation of the CSS still needs to be further investigated, especially regarding module 4, and replication of the CSS of all modules by independent research groups is warranted.

Further, it remains to be clarified which individual factors may influence future ASD severity. Previous studies showed that levels of intelligence and language development were predictive of ASD symptom severity later in life (Billstedt et al. 2005, 2007; Gillberg and Steffenburg 1987; McGovern and Sigman 2005). However, to our knowledge, there is only one study that investigated whether the presence of comorbid psychiatric problems influenced later ASD symptom severity (Simonoff et al. 2013). Although co-occurring psychiatric problems are assumed to be a prognostic factor for ASD (APA 2013), and are assumed to intensify the core symptoms of ASD (Wood and Gadow 2010), the study of Simonoff et al. (2013) found that an increase or decrease of psychiatric problems from 12 to 16 years of age was not associated with ASD symptom severity at the age of 16. These researchers recommended that future studies should use diagnostic interviews rather than questionnaires to further evaluate the association between ASD severity and other psychiatric disorders over time.

Finally, when examining the outcome of individuals with ASD, it is not only important to investigate symptomatology, but also to gain further insight on how symptomatology is impacting daily life by exploring the relation with societal functioning (e.g. participation in education, peer relations etc.). Previous studies on societal functioning of individuals diagnosed with ASD in childhood for instance concluded that only a minority of the individuals with ASD attended mainstream schools without special educational needs (SEN) in adolescence (i.e. between 2 and 44 %; Ballaban-Gil et al. 1996; Billstedt et al. 2011; Cederlund et al. 2008; Farley et al. 2009; Howlin et al. 2004, 2013). Knowledge on the societal functioning of this group might contribute to more optimally tailored services for these individuals.

Taken together, the long-term stability of ASD severity in cognitively able individuals with PDD-NOS, and the association with psychiatric comorbidity and societal functioning, need further investigation using a dimensional approach and state-of-the art assessment methods. Therefore, we conducted a longitudinal study on a sample of cognitively able individuals diagnosed with PDD-NOS in childhood. The first aim of this prospective study was to evaluate the stability of ADOS ASD severity over a 7 years period from childhood to adolescence. The second aim was to examine whether psychiatric comorbidity in childhood was associated with ADOS ASD severity (i.e. CSS) in adolescence. The third aim was to explore the association of ADOS ASD severity in adolescence with societal functioning (i.e. mental health care use, SEN and age-appropriate reciprocal friendships) in adolescence.

## Methods

### Participants

Participants in the current prospective study were 72 cognitively able individuals who received a clinical DSM-IV-TR classification of pervasive developmental disorder-not otherwise specified (PDD-NOS) in childhood. This sample was retrieved as follows: 503 individuals were clinically referred for psychiatric evaluation to the outpatient Department of Child and Adolescent Psychiatry/Psychology of Erasmus MC - Sophia children’s hospital between July 2002 and September 2004. At the time of inclusion, this department was specialized in diagnosing children with suspected ASD presenting with milder or atypical symptoms and with average to high intelligence. As such, this department especially drew referrals of this type, often resulting in a diagnosis of PDD-NOS and no intellectual disability. During the first assessment wave, a diagnostic assessment procedure took place, including parental questionnaires, e.g. the Children’s Social Behavior Questionnaire ([CSBQ] Hartman et al. 2006) and the Child Behavioral Checklist ([CBCL] Verhulst et al. 1996), semi-structured interviews, e.g. the Semi-structured Clinical Interview for Children & Adolescents (Kasius 1997), assessment of early developmental and medical history, psychiatric observation of the child in a one-to-one situation, psychological assessments (e.g. intelligence and neuropsychological tests), and school information. A multi-disciplinary team based their clinical consensus DSM-IV-TR classification on all information obtained during this diagnostic assessment procedure. In total, 114 children met the DSM-IV-TR criteria for a pervasive developmental disorder-not otherwise specified (PDD-NOS). Of these 114 individuals, the 97 children with an Full Scale Intelligence Quotient (FSIQ) of 70 or higher were included in the current study. The parents of these 97 children agreed with further assessment using the Autism Diagnostic Observation Schedule at Wave 1 ([ADOS], Lord et al. 1999), which was performed by a trained and certified psychologist. At Wave 2, approximately 7 years later (mean follow-up time = 6.9, SD = .6), these 97 individuals and their parents were asked to participate in a follow-up study. Of these 97 individuals, 72 individuals and their parents agreed to participate in the ADOS assessment at Wave 2, representing a 74 % follow-up rate. Thus, the participants of the current study were the 72 individuals with PDD-NOS and an FSIQ of 70 or higher, who had complete data on the ADOS both in childhood (Wave 1: mean age = 9.2 years, SD = 1.8; mean FSIQ = 96.4, SD = 14.1) as well as in adolescence (Wave 2: mean age = 16.1 years, SD = 1.9; mean FSIQ = 103.1, SD = 12.6). A Chi square test and *t* tests revealed that the 72 participants (63 boys, 9 girls) did not significantly differ from the individuals who did not participate during the Wave 2 with regard to initial age (t(95) = .03, *p* = .97), gender (χ^2^(1) < .1, *p* = .95), childhood FSIQ (t(95) = −1.9, *p* = .07) or childhood ASD symptom severity (i.e. as measured with the ADOS calibrated severity score; *t*(95) = −.16, *p* = .88). The majority of the sample (99 %) had a Dutch nationality. Also, when considering ethnic background, data concerning the country of birth of the parents of the individuals was reported for 67 participants. Sixty-four (95 %) of the fathers of the participants were born in the Netherlands, 63 (94 %) of the mothers of the participants were born in the Netherlands. With regard to socio-economic status, at the first assessment wave, 43 % of the fathers and 54 % of the mothers completed post-secondary education and the majority of the parents had a paid job (i.e. 92 % of the fathers and 69 % of the mothers).

During Wave 1, parents of the participating children signed informed consent forms prior to participation in the study. During Wave 2, both parents and adolescents signed the informed consent forms. This study was approved by the Medical Ethics Committee of the Erasmus MC (MEC-2008-388).

### Measures

The *Autism Diagnostic Observation Schedule* ([ADOS] Gotham et al. 2007; Lord et al. 2000) was administered to assess ASD severity in childhood (Wave 1) and in adolescence (Wave 2). The ADOS is a semi-structured, standardized assessment tool to evaluate ASD symptoms regarding communication, reciprocal social interaction, and repetitive activities and interests. The ADOS consists of five modules; the selection of the appropriate module is based on the individual’s expressive language skills and chronological age (Gotham et al. 2007; Lord et al. 2000). At Wave 1, module 3 was administered to all participants, while at Wave 2, module 4 was administered to all participants. The ADOS was administered by an examiner who had completed the ADOS Research Training and achieved reliability for administration and coding. The examiners at both assessment waves were blind to all clinical and diagnostic information. The ADOS calibrated severity scores (CSS) were used to examine ADOS symptom severity in childhood and in adolescence (Gotham et al. 2009; Hus et al. 2014). In the current sample, the CSS at Wave 1 (i.e. Module 3) was not associated with age, gender, FSIQ, or non-ASD specific emotional or behavioral problems (i.e. Anxiety disorders, Mood disorders, Developmental disorders or Disruptive disorders as measured with the DISC) at Wave 1 (age; *r* = −.08, *p* = .51, gender; *r*_*b*_ = .23, *p* = .23, FSIQ; *r* = −.08, *p* = .53, Anxiety disorders; *r*_*b*_ = −.08, *p* = .57, Mood disorders; *r*_*b*_ = −.20, *p* = .36, Developmental disorders; *r*_*b*_ = .07, *p* = .67, Disruptive disorders: *r*_*b*_ = −.11, *p* = .51). Also the CSS at Wave 2 (i.e. Module 4) was not associated with age, gender, FSIQ, or non-ASD specific emotional or behavioral problems at Wave 2 (age; *r* = −.15, *p* = .21, gender; *r*_*b*_ = −.09, *p* = .66, FSIQ; *r* = −.19, *p* = .14, Anxiety disorders; *r*_*b*_ = .17, *p* = .28, Mood disorders; *r*_*b*_ = −.05, *p* = .78, Developmental disorders; *r*_*b*_ < −.01, *p* = .98, Disruptive disorders: *r*_*b*_ = .02, *p* = .88).

The *Diagnostic Interview Schedule for Children* ([DISC—parent informant] Schaffer et al. 1998) was used to assess other psychiatric disorders at Wave 1 and at Wave 2. The DISC is a fully structured parental interview assessing all common DSM-IV-TR (APA 2000) disorders in children and adolescents (Shaffer et al. 1998). The DISC-IV inquires about thirty-four diagnoses in twenty-six diagnostic sections. In the current study the following broad categories and underlying classifications were considered: 1) Anxiety Disorders; social phobia, separation anxiety disorder, specific phobia, panic disorder without and with agoraphobia, generalized anxiety disorder, selective mutism, obsessive–compulsive disorder, and posttraumatic stress disorder, 2) Mood disorders; major depression, dysthymic disorder, mania, hypomania, 3) Developmental disorders; attention deficit hyperactivity disorder (ADHD) predominantly inattentive type, ADHD predominantly hyperactive-impulsive type, ADHD combined type, 4) Disruptive disorders; oppositional defiant disorder and conduct disorder. For each disorder, parents were asked questions concerning symptom criteria. The majority of these questions are answered in a ‘yes’ or ‘no’ fashion. Algorithms were applied to evaluate whether the child met the criteria for a classification for the above mentioned disorders (Schaffer et al. 2000). The DISC was developed as a categorical measure, resulting in outcomes regarding the absence or presence of a disorder, with DISC classifications coded as either 0 (i.e. absent) or 1 (i.e. present). In addition, we calculated the total number of DISC classifications (ranging from 0 up to 15, given the total number of classifications that were assessed, and ranging from 0 to 9 in the current data set). The DISC is commonly used in samples with typically developing individuals, showing adequate interrater and test–retest reliability (Schaffer et al. 2000) and it has been used in two ASD-samples to assess comorbid psychiatric disorders (de Bruin et al. 2007; Muris et al. 1998).

To generate *Full Scale Intelligence Quotient* (*FSIQ*) scores, the Wechsler Intelligence Scale for Children-Revised (WISC-R, Wechsler 1974) was administered at Wave 1, and the Wechsler Abbreviated Scale of Intelligence (WASI, Wechsler 1999) was administered at Wave 2.

### Societal Functioning

Societal functioning, i.e. the impact on functioning in daily life, was operationalized as 1) mental health care use, 2) special educational needs (SEN), and 3) age appropriate, reciprocal friendships.

To assess *mental health care use* between Wave 1 and 2, a questionnaire concerning health care use was filled-out by the parents at Wave 2. This questionnaire was similar to a questionnaire that was used in the Tracking Adolescents’ Individual Lives Survey (TRAILS; Amone-P’Olak et al. 2010), a prospective cohort study of Dutch adolescents from the general population that are followed into adulthood. At Wave 2, parents filled-out nine items on whether their children received mental health care interventions (e.g. ‘did your child visit a psychologist’, and ‘did your child visit an institution for mental health care’) for the period between Wave 1 and Wave 2. Items were scored as 0 (i.e. not used) or 1 (i.e. used). The nine items were combined into one dichotomous variable, with a score of 0 indicating no use of mental health care, and a score of 1 indicating the use of mental health care between the two assessment waves.

Information concerning *the type of education* (i.e. *with or without special educational needs [SEN]*) was obtained as part of the Autism Diagnostic Interview-Revised ([ADI-R]; e.g. section ‘background questions’, Rutter et al. 2003) which was administered in adolescence (Wave 2) in the majority of parents (n = 69). The ADI-R was assessed at Wave 2 by an examiner who had completed the ADI-R Research Training and achieved reliability for administration and coding, and who was blind for all other diagnostic information. In the Netherlands, children with SEN can either attend a mainstream school with or without on-site extra guidance, or they can attend education in a specialized school (Stoutjesdijk and Scholte 2009). In the current study, adolescents that received SEN (i.e. either in regular schools or in special schools) were coded as 1, adolescents that attended regular schools without SEN were coded as 0.

To obtain an index on whether the adolescent currently had any *age*-*appropriate reciprocal friendship*, as reported by their parents, the item concerning friendship on the ADI-R (i.e. item number 65) was used. For the current purpose, the four answer categories on the ADI-R friendships item were recoded into two categories; ‘no reciprocal friendship’ (i.e. a score of 1, 2, or 3 on the friendship item) or ‘age-appropriate reciprocal friendship’ (i.e. a score of 0 in the friendship item).

### Statistical Analyses

Firstly, descriptive statistics were calculated for the total sample, and stratified for individuals with stable, increased or decreased ASD symptom severity (ADOS CSS) from childhood to adolescence.

Subsequently, to evaluate stability of ASD symptom severity (aim 1), a bivariate correlation, and an intraclass correlation, between Wave 1 ADOS CSS and Wave 2 ADOS CSS were calculated. To further determine the stability of ASD symptom severity, a Reliable Change Index (RCI; Zahra and Hedge 2010) was calculated for each participant, based on the ADOS CSS in childhood and adolescence. An RCI with a magnitude of ±1.96 was considered significant, in line with Zahra and Hedge 2010. This resulted in three groups: a) individuals who showed stable ASD symptom severity (i.e. an RCI with a magnitude between −1.96 and +1.96, i.e. less than 2 points difference is CSS), b) individuals who showed an increase (i.e. two or more points increase in their CSS) in ASD symptom severity, and c) individuals who showed a decrease (i.e. two or more points decrease in their CSS) in ASD symptom severity. To evaluate the stability of ADOS ASD *classifications*, proportions of individuals with different a developmental course were calculated, i.e. (a) those who remained to fulfil the criteria (i.e. ADOS diagnostic algorithm) for an ADOS ASD classification in adolescence, (b) those who no longer met the criteria for an ADOS classification for ASD in adolescence (c) those who did not meet criteria for an ADOS classification of ASD in childhood and in adolescence, and (d) those who did not meet criteria for an ADOS classification in childhood, but who did in adolescence.

To evaluate the association of childhood characteristics (age, gender, FSIQ, ASD symptom severity and comorbid psychiatric disorders) with ASD symptom severity in adolescence (i.e. T2 ADOS CSS) (aim 2), we planned to perform a multiple regression analysis. To examine which variables needed to be included in the multiple regression analysis, we preliminary calculated the correlations between all putative predictor variables (i.e. age, gender, Wave 1 intelligence, Wave 1 ADOS CSS and Wave 1 comorbid psychiatric disorders [i.e. anxiety disorder, mood disorder, ADHD and disruptive disorder] in childhood), and the outcome variable (i.e. ADOS CSS in adolescence); we calculated Pearson correlations if both variables were continuous and biserial correlations if one of the variables was a dichotomous. In order to maintain optimal power, the predictor variables were only included in the multivariate model if they correlated significantly with the outcome variable. Similar analyses were performed with the RCI as the outcome measure, to evaluate whether childhood variables predicted *change in* ASD symptom severity.

Finally, to examine the association of ASD symptom severity with societal functioning (aim 3), descriptive statistics were provided and biserial correlations were calculated between societal functioning indices (i.e. mental health care use, SEN and age-appropriate, reciprocal friendships) and Wave 2 ADOS CSS. Similar analyses were performed with the RCI as the outcome measure, to examine whether these indices were associated with *change in* ASD severity. As part of these analyses, we explored the association of the societal functioning indices with age, gender and FSIQ. If these characteristics were significantly associated with ASD symptom severity, multiple regression analysis were performed including these variables as covariates.

The variables associated with the independent variable (i.e. ASD symptom severity) were empirically selected, therefore we performed no correction for multiple testing.

## Results

### Stability of ASD

**Fig. 1:**
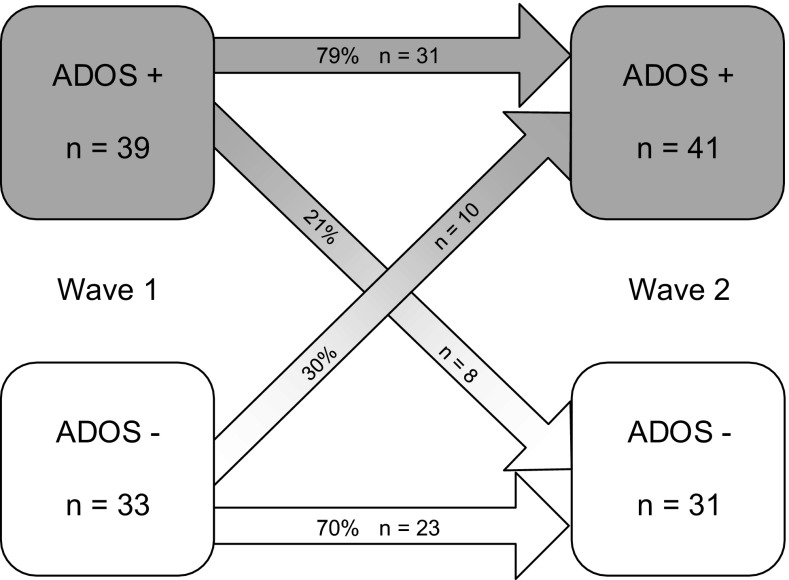
Stability of ADOS classifications form childhood (Wave 1) to adolescence (Wave 2)

The bivariate correlation between the Wave 1 ADOS CSS and the Wave 2 ADOS CSS was .51 (*p* < .001) with an OR 8.9, meaning that the odds of an ADOS ASD classifications was 8.9 times higher for individuals with an ADOS ASD classification at Wave 1 compared to individuals without an ADOS ASD classification at Wave 1 (95 % CI 3.0–26.1).The intraclass correlation (ICC) was .65 (*p* < .001). The Reliable Change Index (RCI) resulted in information concerning the stability of *ASD symptom severity* (e.g. based on the ADOS CSS) from childhood to adolescence. In 40 % (n = 29) of the individuals in the current sample the ASD symptom severity increased significantly (i.e. more than two points increase in the CSS), in 20 % (n = 14) of the individuals the ASD symptom severity decreased significantly (i.e. more than two points decrease in the CSS). The ASD symptom severity of the other 40 % (n = 29) of the individuals within this group did not significantly change from childhood to adolescence (i.e. less than two points change in the CSS). Descriptive statistics for these three groups are presented in Tables 1 and 2. The stability of *ASD classifications* (e.g. based on the ADOS diagnostic algorithm classifications) is illustrated in Fig. 1. Seventy-nine percent (n = 31) of the individuals with an ADOS ASD classification in childhood (i.e. ADOS +) also received an ADOS ASD classification in adolescence. Thus, 21 % (n = 8) of the individuals with an ADOS ASD classification in childhood, no longer met the criteria for an ADOS ASD classification in adolescence. Seventy percent (n = 23) of the individuals without an ADOS ASD classification in childhood (i.e. ADOS -) did also not meet criteria for an ADOS ASD classification in adolescence. Thirty percent (n = 10) of the individuals not meeting ADOS ASD criteria in childhood did meet these criteria in adolescence.

**Table 1:** Childhood characteristics (Wave 1) of individuals with stable, increased and decreased ASD symptom severity (ADOS CSS) from childhood to adolescence

Childhood characteristics	Total (n = 72)	Stable ADOS CSS (n = 29)	Increase ADOS CSS (n = 29)	Decrease ADOS CSS (n = 14)
Age, *Mean* (*SD*)	9.2 (1.8)	9.1 (1.8)	9.1 (1.8)	9.6 (1.9)
Gender, *% boy*	88 %	83 %	86 %	100 %
FSIQ, *Mean* (*SD*)	96.4 (14.1)	95.9 (13.3)	94.8 (14.1)	100.8 (15.8)
SA CSS, *Mean* (*SD*)	4.5 (2.4)	4.1 (2.5)	4.2 (2.2)	6.0 (1.9)
RRB CSS, *Mean* (*SD*)	4.8 (2.7)	4.5 (2.8)	4.7 (2.7)	5.6 (2.4)
Anxiety disorder^a^, *% present*	49 %	46 %	59 %	36 %
Mood disorder^a^, *% present*	9 %	15 %	7 %	0 %
Developmental disorder^a^, *% present*	45 %	46 %	48 %	36 %
Disruptive disorder^a^, *% present*	27 %	27 %	26 %	29 %
# psychiatric disorders^a^, *Mean* (*SD*)	1.7 (1.6)	1.8 (1.5)	1.8 (1.8)	1.2 (1.4)

The psychiatric disorders are based on the Diagnostic Interview Schedule for Children (DISC)

*ADOS CSS* Autism Diagnostic Observation Schedule, calibrated severity score, *FSIQ* Full Scale Intelligence Quotient, *SA CSS* Social Affect calibrated severity score, *RRB CSS* restricted repetitive behaviour calibrated severity score

* *p* < .05; ** *p* < .01

^a^DISC data is missing in n = 5

**Table 2:** Characteristics in adolescence (Wave 2) of individuals with stable, increased and decreased ASD symptom severity (ADOS CSS) from childhood to adolescence

Adolescence characteristics	Total (n = 72)	Stable ADOS CSS (n = 29)	Increase ADOS CSS (n = 29)	Decrease ADOS CSS (n = 14)
Age, *Mean* (*SD*)	16.1 (1.9)	16.1 (2.0)	16.0 (1.9)	16.4 (1.9)
FSIQ, *Mean* (*SD*)	103.1 (12.6)	100.6 (15.1)	103.1 (9.4)	107.8 (12.3)
SA CSS, *Mean* (*SD*)	5.2 (2.7)	4.3 (2.3)	7.2 (1.9)	3.1 (2.0)
RRB CSS, *Mean* (*SD*)	5.7 (1.8)	5.6 (.8)	6.5 (1.8)	4.5 (2.4)
Anxiety disorder^a^, *% present*	35 %	37 %	45 %	8 %
Mood disorder^a^, *% present*	13 %	19 %	10 %	8 %
Developmental disorder^a^, *% present*	38 %	37 %	45 %	23 %
Disruptive disorder^a^, *% present*	26 %	22 %	35 %	15 %
# psychiatric disorders^a^, *Mean* (*SD*)	1.3 (1.7)	1.4 (1.9)	.5 (1.0)	1.6 (1.7)
Mental health care use^b^, *% users*	87 %	85 %	89 %	83 %
Special Educational Needs, *% users*	71 %	74 %	78 %	50 %
Friendship, *% present*	16 %	21 %	4 %	21 %

The psychiatric disorders are based on the Diagnostic Interview Schedule for Children (DISC)

*ADOS CSS* Autism Diagnostic Observation Schedule, calibrated severity score, *FSIQ* Full Scale Intelligence Quotient, *SA CSS* Social Affect calibrated severity score, *RRB* *CSS* restricted repetitive behaviour calibrated severity score

* *p* < .05; ** *p* < .01

^a^DISC data is missing in n = 3

^b^Mental health care between the first and the second assessment wave

### Relations of Childhood Characteristics and Comorbid Psychiatric Disorders with ASD Severity in Adolescence

As shown in Table 3, childhood characteristics (age, gender, FSIQ and all types of comorbid psychiatric classifications) were not significantly associated with ASD symptom severity in adolescence. Also, the childhood characteristics and all types of comorbid psychiatric disorders were not significantly associated with *the change in* ASD symptom severity from childhood to adolescence (i.e. RCI).

**Table 3:** Correlations of childhood characteristics (age, gender, FSIQ, ASD symptom severity, psychiatric disorders) and ASD symptom severity in adolescence

	Wave 1 age	Wave 1 gender	Wave 1 FSIQ	Wave 1 ASD symptom severity	Wave 1 Anxiety disorders	Wave 1 Mood disorders	Wave 1 Developmental disorders	Wave 1 Disruptive disorders	Wave 1 # psychiatric disorders
Wave 2 ASD symptom severity	−.16	−.08	−.23	.51**	.04	−.11	.28	−.04	.04
RCI	.10	.31	.19	.33**	−.13	−.05	−.24	−.05	−.14

The psychiatric disorders are dichotomous variables based on the Diagnostic Interview Schedule for Children (DISC). *RCI* Reliable Change Index: change in CSS between Wave 1 and Wave 2, *ASD symptom severity* Autism Spectrum Disorder symptom severity: based on the *ADOS CSS* Autism Diagnostic Observation Schedule, calibrated severity score, *IQ* Intelligence Quotient

* *p* < .05; ** *p* < .01

### Relations of ASD Severity in Adolescence with Societal Functioning in Adolescence

As shown in Table 2, mental health care use between the first and the second assessment wave was high (87 %). Also, special educational needs were high (71 %). Sixty percent of the individuals with SEN were placed in special schools, and 40 % of the individuals with SEN were placed in main stream schools, but received additional help. Parents reported that only 16 % of the adolescents with a PDD-NOS classification in childhood had at least one age-appropriate, reciprocal friendship during adolescence. Mental health care use between childhood and adolescence was not significantly associated with ASD symptom severity in adolescence (Mental health care use; *r*_b_ = −.06, OR .96 [95 % CI .74–1.24], *p* = .74). SEN in adolescence was significantly associated with ASD symptom severity in adolescence (*r*_b_ = −.44, OR .75 [95 % CI .60–.94), *p* = .01). Adolescents with a higher level of ASD symptom severity were more likely to have SEN. Also, adolescents with a higher level of ASD symptom severity were less likely to have age-appropriate reciprocal friendships, as reported by their parents (*r*_b_ = .45, OR 1.43 [95 % CI 1.07–1.90, *p* = .01). The societal functioning variables were not significantly associated with the RCI variable. Age, gender and FSIQ at Wave 2 were not significantly associated with the societal functioning indices.

## Discussion

In the current prospective study, we examined the 7-year stability of clinically observed ASD symptom severity, as well as its relation with psychiatric comorbidity, and with societal functioning, in a sample of 72 cognitively able individuals diagnosed with PDD-NOS in childhood.

Previous studies concerning the stability of ASD mostly included cases with more severe ASD, used a categorical approach towards ASD, and found a high diagnostic stability (for a review see: Woolfenden et al. 2012). The newly introduced calibrated severity score (CSS) of the ADOS creates the possibility to examine ASD stability using a dimensional approach, while using information from clinical observation. Previous studies have indicated that CSS are less influenced by participant demographics and non-ASD specific behavior problems than raw total scores on the ADOS (Gotham et al. 2009; Hus and Lord 2014; Bal and Lord 2015). This finding was replicated in the current study (i.e. including module 4 assessments). We found a correlation between ADOS calibrated severity scores in childhood and adolescence of .51, indicating a large 7-year stability of ASD symptom severity (Cohen 1988). This temporal stability is almost comparable with the stability found for the symptom severity of other childhood psychiatric problems (i.e. a correlation of .39 for internalizing problems and a correlation of .50 for externalizing problems for an 8 year follow-up, Verhulst and Van der Ende 2013). By presenting this 7-year stability of ASD severity, and comparing it to the similar stability of externalizing psychiatric symptom severity, we hope to raise the awareness that, although ASD is generally considered a ‘life-long’ condition, alterations in the severity of core symptoms seem to occur over time to a similar degree as in externalizing psychiatric problems. It is important to note that 40 % (n = 29) of the individuals diagnosed with PDD-NOS in our sample showed a significant increase in ASD severity. So although individuals with a diagnosis of PDD-NOS might be regarded as ‘mild cases’ in childhood, a noticeable proportion may go on to develop more, or more severe, symptoms later in life, warranting further attention. Also, 20 % of the individuals diagnosed with PDD-NOS in childhood showed a decrease in symptom severity. When the stability of the ADOS ASD classifications was evaluated categorically, we found that the majority of individuals had stable ADOS classifications. However, 21 % (n = 8) seemed to no longer meet criteria for an ADOS ASD classification. Also when considering the clinical best estimate diagnoses, 29 % of the individuals diagnosed with PDD-NOS in childhood no longer met criteria for an ASD diagnosis in adolescence. Individuals who no longer meet the diagnostic criteria of ASD later in life, have been referred to as ‘Optimal Outcome (OO)’ in previous studies (Fein et al. 2013; Granpeesheh et al. 2009; Helt et al. 2008; Orinstein et al. 2015). The review of Helt et al. (2008) indicated that 3–25 % of the individuals with ASD in childhood lost their ASD diagnosis later in life and fell within the normal range of cognitive and adaptive functioning. Comorbid psychiatric disorders were frequently present later in life (Fein et al. 2005; Helt et al. 2008). Also in the current sample, a large proportion (i.e. 62 %) of the group that no longer met criteria for an ASD diagnosis did meet criteria for another psychiatric diagnosis in adolescence, which puts the term ‘optimal outcome’ in a more nuanced perspective (data available upon request). Shifts in primary diagnostic classification seem to occur, which warrants follow-up assessments later in life of individuals diagnosed with PDD-NOS in childhood.

Since the current sample only included individuals with PDD-NOS and an IQ above 70, one might wonder whether the current findings apply to individuals diagnosed with ASD according to the DSM-5. The participants in the current sample were also included in a larger study on phenotypic profiles of children with PDD (Greaves-Lord et al. 2012). Greaves-Lord et al. (2012) indeed found that the phenotypic profile of individuals with PDD and an IQ above 70 was not fully alike the conceptualization of ASD in the DSM-5. About 30 % of the individuals with PDD-NOS and an IQ above 70 showed a profile which seemed to be more in line with the DSM-5 classification Social (Pragmatic) Communication Disorder. Thus, the presented data gives some insight in the long-term stability of individuals diagnosed with ASD before the introduction of the DSM-5, which might provide insight in these outcomes for a large group of individuals diagnosed with PDD-NOS in the last decade. However, these finding might not apply to all children diagnosed with ASD today (i.e. according to the DSM-5). The reader should interpret the current findings against this background.

Previous research has suggested that differences in ASD symptom severity might be related to comorbid psychiatric disorders (Wood and Gadow 2010). In the current follow-up study, psychiatric comorbidities (i.e. the presence or absence of anxiety disorder, mood disorder, developmental disorder and disruptive disorder) in childhood and in adolescence were not significantly associated with ASD severity in adolescence. Simonoff et al. 2013 also investigated similar associations—however using continuous measures—and showed no association of increased or decreased comorbid psychiatric symptoms between childhood and adolescence with ASD severity in adolescence. Thus, although high comorbidity rates have often been reported in ASD in both the current study and in previous studies (de Bruin et al. 2007; Matson and Nebel-Schwalm 2007; Simonoff et al. 2008; van Steensel et al. 2013), developmental trajectories of ASD and comorbid diagnoses might not be as clearly intertwined as assumed (Lecavalier et al. 2009). The current findings are not in line with previous cross-sectional studies, since previous studies did find associations between comorbid psychiatric symptoms and ASD symptoms. For example, higher levels of anxiety in individuals diagnosed with ASD were associated with lower quality of social relations (Eussen et al. 2013) and more social problems (Dubin et al. 2015). Clearly, more research regarding the longitudinal relationship between comorbid psychiatric symptoms and ASD symptoms is needed, since definite conclusions cannot be drawn based on the few longitudinal studies conducted so far.

Besides the stability of ASD symptoms and the relation to comorbid disorders, societal functioning, i.e. the impact of ASD on functioning in daily life in adolescence, was investigated. The societal burden in adolescence seemed substantial, since the majority of the participants received professional mental health care between childhood and adolescence (i.e. 87 %), and had special educational needs (SEN; i.e. 71 %). These high levels of mental health care use and SEN between childhood and adolescence might not be surprising, since individuals referred for diagnostic assessment very often are referred due to supposed mental health care and educational needs. The percentage of individuals that had SEN is comparable to previous research that included individuals with ASD without a cognitive impairment (between 56 and 72 %; Cederlund et al. 2008; Farley et al. 2009; Howlin et al. 2013), but the currently reported percentage of SEN is substantially lower than that in other studies that included individuals diagnosed with ASD with a cognitive impairment (between 85 and 98 %; Ballaban-Gil et al. 1996; Billstedt et al. 2011; Howlin et al. 2004). The percentages of mental health care and SEN in individuals with ASD are much higher than numbers from the general population; i.e. in which only 12 % of the individuals of 10–20 years old used mental health care (CBS 2010) and only 5 % of the adolescents of 12–18 years old needed special education (European Agency for Development in Special Needs Education 2010). Mental health care use was not significantly associated with ADOS ASD symptom severity. In other words, mental health care use was high, regardless of the severity of core ASD symptoms. SEN were associated with ASD symptom severity, so adolescents with a lower ASD symptom severity less often had SEN. Also, the level of ASD symptom severity was associated with the absence or presence of at least one age-appropriate, reciprocal friendship. The parents of adolescents with higher levels of ASD symptom severity were more likely to report that their child did not have a reciprocal friendship. Therefore, overall, the burden on societal functioning in adolescence can be considered substantial for cognitively able individuals diagnosed with PDD-NOS in childhood.

The current findings concerning the stability of ASD symptom severity, the limited relation with psychiatric comorbidity, as well as the societal impact of ASD in adolescence, may help professionals, parents and policy makers to better understand, and cope with, the long-term prospects of cognitively able children diagnosed with PDD-NOS in the previous decade. Overall, changes in ASD symptom severity seem to occur, and comorbid psychiatric disorders were common both in childhood and adolescence in all individuals with PDD-NOS. Therefore, in clinical practice, follow-up assessment later in life, reevaluating ASD symptom severity as well comorbid conditions, seems useful in cognitively able individuals who are diagnosed with PDD-NOS in childhood.

A limitation of the current study is that only referrals the one university center were included, therefore the population is not representative of other clinical populations. Also, use of the ADI-R at Wave 1 would have improved the diagnostic information on our sample. However, the overall use of well-validated, quantitative standardized measures that are widely used internationally, does provide the possibility to translate the current results to other clinical settings. Notwithstanding the limitations of the current study, we hope that the current findings will trigger further research into the interrelations among the developmental trajectories of ASD, comorbid psychiatric diagnoses and their impact on societal functioning.
